# In Vitro Antioxidant, Antitumor and Photocatalytic Activities of Silver Nanoparticles Synthesized Using *Equisetum* Species: A Green Approach

**DOI:** 10.3390/molecules26237325

**Published:** 2021-12-02

**Authors:** Denisa Batir-Marin, Cornelia Mircea, Monica Boev, Ana Flavia Burlec, Andreia Corciova, Adrian Fifere, Alexandra Iacobescu, Oana Cioanca, Liliana Verestiuc, Monica Hancianu

**Affiliations:** 1 Department of Pharmaceutical Sciences, Faculty of Medicine and Pharmacy, Dunarea de Jos University, 800010 Galati, Romania; denisa.batir@ugal.ro (D.B.-M.); monica.boev@ugal.ro (M.B.); 2 Department of Pharmaceutical Biochemistry and Clinical Laboratory, Faculty of Pharmacy, “Grigore T. Popa” University of Medicine and Pharmacy, 16 University Street, 700115 Iasi, Romania; corneliamircea@yahoo.com; 3 Department of Drug Analysis, Faculty of Pharmacy, “Grigore T. Popa” University of Medicine and Pharmacy, 16 University Street, 700115 Iasi, Romania; 4 Centre of Advanced Research in Bionanoconjugates and Biopolymers Department, “Petru Poni” Institute of Macromolecular Chemistry, 41A Grigore Ghica Voda Alley, 700487 Iasi, Romania; fifere@icmpp.ro (A.F.); iacobescu.alexandra@icmpp.ro (A.I.); 5 Department of Pharmacognosy, Faculty of Pharmacy, “Grigore T. Popa” University of Medicine and Pharmacy, 16 University Street, 700115 Iasi, Romania; oana.cioanca@gmail.com (O.C.); mhancianu@yahoo.com (M.H.); 6 Department of Biomedical Sciences, Faculty of Medical Bioengineering, “Grigore T. Popa” University of Medicine and Pharmacy, 16 University Street, 700115 Iasi, Romania; liliana.verestiuc@umfiasi.ro

**Keywords:** *Equisetum pratense*, *Equisetum telmateia*, *Equisetum sylvaticum*, silver nanoparticles, green synthesis, photocatalytic activity, antioxidant capacity, cytotoxicity

## Abstract

The ethanolic extracts of three *Equisetum* species (*E. pratense* Ehrh., *E. sylvaticum* L. and *E. telmateia* Ehrh.) were used to reduce silver ions to silver nanoparticles (AgNPs). The synthesized AgNPs were characterized using UV-Vis spectrophotometry, Fourier Transform Infrared Spectroscopy (FTIR), Energy Dispersive X-ray (EDX), Transmission Electron Microscopy (TEM) and Dynamic Light Scattering (DLS) measurements. FTIR data revealed the functional groups of biomolecules involved in AgNPs synthesis, such as O-H, C-H, C=O, C-O, and C-C. EDX spectroscopy was used to highlight the presence of silver, while DLS spectroscopy provided information on the mean diameter of AgNPs, that ranged from 74.4 to 314 nm. The negative Zeta potential values (−23.76 for Ep–AgNPs, −29.54 for Es–AgNPs and −20.72 for Et–AgNPs) indicate the stability of the obtained colloidal solution. The study also focused on establishing the photocatalytic activity of AgNPs, which is an important aspect in terms of removing organic dyes from the environment. The best photocatalytic activity was observed for AgNPs obtained from *E. telmateia*, which degraded malachite green in a proportion of 97.9%. The antioxidant action of the three AgNPs samples was highlighted comparatively through four tests, with the best overall antioxidant capacity being observed for AgNPs obtained using *E. sylvaticum*. Moreover, the biosynthesized AgNPs showed promising cytotoxic efficacy against cancerous cell line MG63, the AgNPs obtained from *E. sylvaticum* L. providing the best result, with a LD_50_ value around 1.5 mg/mL.

## 1. Introduction

The potential use of nanomaterials is a continuously developing research field. Metal nanoparticles, in particular, have received special attention due to their widespread applications in areas such as medicine, electronics, cosmetics and food, optics, chemical industry and many others [1,2].

Among metal nanoparticles, silver nanoparticles (AgNPs) have unique properties such as chemical stability, catalytic activity, antibacterial, antiviral, antifungal, anti-inflammatory and anticancer properties, which makes them medically relevant [3,4].

In general, for AgNPs synthesis, a variety of chemical, physical and biological methods can be used. Biological methods currently represent the center of attention for researchers, since they provide numerous advantages, given that they involve eco-friendly processes, low costs and offer the possibility of avoiding high temperatures and pressure. Therefore, the current trend in AgNPs synthesis is the use of microorganisms, enzymes, and plants extracts. However, plants are usually preferred given their high accessibility and the presence of biological compounds that can participate in this process, as well as the feasibility of the synthesis process [5,6].

The existing literature contains numerous examples of plant extracts that can be used to synthesize AgNPs, which belong to families such as: *Acanthaceae, Amaranthaceae, Apocynaceae, Asphodelaceae, Asteraceae, Burseraceae, Dioscoreaceae, Euphorbiaceae, Fabaceae, Lamiaceae, Moraceae, Myrtaceae, Poaceae, Rutaceae*, and *Solanaceae* [6,7].

Compounds involved in AgNPs synthesis are distributed in different parts of the plant (leaves, fruits, seeds, roots) and consist of primary (carbohydrates, proteins, peptides, amino acids, vitamins) and secondary (alkaloids, flavonoids, terpenoids, phenolic acids) plant metabolites [8,9].

Biological actions of AgNPs depend on the size, particle morphology and composition, coating, agglomeration tendency, particle reactivity in solution, ion release efficiency, cell type, as well as on the reducing agent used for the obtaining of particles [10]. Studies in rats have shown that AgNPs synthesized using plant extracts have the capacity of reducing local inflammation in the colon and increase the healing rate of mucosal lesions by stimulating fibroblasts and reducing the concentration of pro-inflammatory cytokines [11]. The antioxidant activity of nanoparticles can also be accompanied by the antiangiogenetic effect obtained through endothelial growth factor inhibition, which can thus reduce the development of tumor processes. The phenomenon has been demonstrated for AgNPs obtained using *Achillea biebersteinii* flower extract [12].

Other studies have shown that nanoparticles can induce cancer cell apoptosis and oxidative stress by mitochondrial damage only in cancerous cells without affecting healthy ones, capture and adsorb cytoplasmic proteins, influence gene transcription and increase the sensitivity of cancerous cells to conventional chemotherapeutics [13].

Cell culture studies have shown that AgNPs are able to induce cytotoxicity in human cell lines including human bronchial epithelial cells, red blood cells, macrophages, liver cells and bone cells especially for a particle size of less than 10 nm. The cytotoxicity of AgNPs has been reported to be dose, size and time-dependent [14].

The *Equisetaceae* family, traditionally known as Horsetails, represents a group of perennial plants that consist of a fertile stem with brown sprouts that develops in early spring and a sterile one, that grows in late spring and persists until late autumn, the latter one being of medical importance [15]. The *Equisetum* genus grows in different regions of Europe, as well as in North, Central and South America [16]. Species of this genus have been used in the traditional medicine of many countries as treatment for tuberculosis, kidney catarrh, pulmonary and gastric hemorrhages, rheumatic diseases, gout, poorly healing wounds and fractures [17,18]. Active compounds found in such species include flavonoids, quercetin glycosides, phenolic acids, alkaloids, equisetonin, phytosterols, isofucosterol, campesterol and tannins [19,20].

The most known and researched species of the *Equisetum* genus is *E. arvense*, for which many biological activities have been confirmed, especially with applications in kidney disorders. Nonetheless, other species of the genus have been popularly used in different diseases. The few studies that focused on investigating other species of *Equisetum* genus emphasized their promising biochemical potential [21].

Therefore, the numerous applications of AgNPs in different fields and the advantages that plants from the *Equisetum* genus have to offer in their synthesis represent the starting point for our research. The purpose of the study is to investigate the potential use of some Horsetails species in the synthesis of AgNPs, that can offer applications in various domains, such as medicine (through the possible antioxidant and antitumor activities), as well as for environment protection through photocatalytic activity.

Therefore, the present study focused on structural and morphological characterization of AgNPs obtained using ethanolic extracts of three less investigated *Equisetum* species: *E. pratense* Ehrh. (Ep), *E. sylvaticum* L. (Es) and *E. telmateia* Ehrh. (Et). To the best of our knowledge, this is the first report on AgNPs obtained using either of the three *Equisetum* species, which emphasizes the novelty of the research.

The analyses were carried out using various techniques such as UV-Vis spectrophotometry, Fourier Transform Infrared Spectroscopy (FTIR), Energy Dispersive X-ray (EDX), Transmission Electron Microscopy (TEM) and Dynamic Light Scattering (DLS) measurements. Following the synthesis of AgNPs using extracts of *Equisetum* species, the photocatalytic action was then tested. Moreover, the antioxidant and antitumor capacities of the synthesized AgNPs were also evaluated comparatively.

## 2. Materials and Methods

### 2.1. Plant Material

*E. pratense* and *E. sylvaticum* were collected from the North-Eastern region of Romania in June–July 2017. *E. telmateia* was harvested in July 2018, also from North-Eastern Romania. The plants were authenticated using fresh material in the Plant Biology Laboratory of the Biology Faculty, “Al. I. Cuza” University, Iasi, Romania. After identification, the plants were dried in shade at a controlled temperature of 23 °C for 21 days, until the mass became constant. Ethanolic extracts were obtained using 10 g dry plant material mixed with 70% ethanol. The samples were placed in the ultrasonic bath at 30 °C for 30 min. The mixture was filtered and then the ethanolic extracts were evaporated using a rotavapor until the mass became constant and no residual water was left. The dried extracts were maintained in a dry, dark place at a temperature of 4 °C until further analysis [22,23].

### 2.2. Green Synthesis of AgNPs

For AgNPs synthesis, the resuspended extracts were added to the AgNO_3_ solution under magnetic stirring. In order to study the synthesis conditions, reaction parameters such as silver nitrate solution concentrations (1, 3, and 5 mM), extract:AgNO_3_ ratios (9:1, 5:5, 1:9), pH values (2, 6, 8) and stirring intervals (0, 30, 60, 120, 180, 240, 300 min) were varied. After establishing the reaction conditions, the obtained colloidal solutions were centrifuged at 10,000 rpm for 30 min. In order to purify the nanoparticles, the supernatant was removed and the AgNPs were redispersed in distilled water, centrifuged and eventually separated [24,25]. This operation was repeated twice, and the obtained AgNPs were dried in an oven at a temperature of 50 °C, until a constant mass was obtained. The synthesized samples were kept at 4 °C and were assigned the following codes: Ep–AgNPs for *E. pratense,* Es–AgNPs for *E. sylvaticum*, and Et–AgNPs for *E. telmateia.*

### 2.3. Physico-Chemical Characterization of the Obtained AgNPs

The formation of AgNPs was observed through the color change of *Equisetum* extracts after being mixed with AgNO_3_. The UV-Vis spectra were recorded in the 300–600 nm range to distinguish the maximum surface plasmon resonance (SPR), using a Jasco V-530 UV-Vis double beam spectrophotometer for this determination. The FTIR spectra were obtained with a Bruker Vertex 70 spectrophotometer, by potassium bromide tableting, over a scan interval of 4000–310 cm^−1^. The EDX qualitative analysis was performed using a Quanta 200 Environmental Scanning Electron Microscope (ESEM) with EDX. The used EDX detector allowed rapid determination of the elementary composition of nanoparticles. The DLS measurements were performed using a Delsa Nano Submicron Particle Size Analyzer (Beckman Colter) that provided the average diameter of nanoparticles and the Zeta potential value. Additionally, TEM analysis was performed using a Hitachi High-Tech HT 7700 Transmission Electron Microscope.

Moreover, prior to the purification and characterization of AgNPs, the supernatant and the initial plant extract were analyzed taking into account the phenolic content using the modified spectrophotometric Folin-Ciocâlteu method [26]. Gallic acid was used as the standard and the results were expressed in mg gallic acid equivalents (GAE)/mL sample.

### 2.4. Photocatalytic Activity

The photocatalytic activity of the synthesized AgNPs was studied by observing the degradation of malachite green under the influence of sunlight at room temperature and under continuous stirring. The color change was monitored over time and samples were taken at certain intervals and centrifuged. The supernatant was scanned in the 500–700 nm range [27,28,29]. Simultaneously, a control that contained solely malachite green stirred under sunlight was also subjected to the determination.

### 2.5. Biological Evaluation Methods

#### 2.5.1. Antioxidant Activity Assays

The following tests were used to study the antioxidant activity: chelation of ferrous ions, LOX inhibition, the hydroxyl radical and superoxide anion radical scavenger capacities. The chelating capacity of ferrous ions was determined using ferrozine which forms a pink complex with maximum absorbance at 562 nm [30]. The determination of the LOX inhibition capacity could be measured given that the active compounds present in the samples inhibit 15-LOX by blocking linoleic acid oxidation and reduce the absorbance measured at 234 nm [31]. The determination of the hydroxyl radical scavenging activity is based on the method in which the hydroxyl radical, formed in the reaction between ferrous ions and hydrogen peroxide, will hydroxylate salicylic acid to form a pink-violet compound with maximum absorbance at 562 nm [32]. The superoxide anion radical scavenging capacity could be determined because the superoxide radical generated by the reduced nicotinamide adenine nucleotide-phenazine methosulfate system reduces nitro blue tetrazole to a violet-blue formazan with a maximum absorbance at 560 nm [33]. For these tests, gallic acid was used as standard. All experiments were performed in triplicate and the mean and standard deviation were calculated. The interference of AgNPs was eliminated by reading for each concentration the absorbance of the sample against a blank that does not contain any color reagents.

#### 2.5.2. In Vitro Cytotoxic Efficacy of AgNPs

The cytocompatibility of AgNPs was evaluated using cell cultures (MG63 osteosarcoma cells) in 96-well microtiter plates, each being populated with 5 × 10^3^ cells, in Dulbecco’s Modified Eagle’s Medium (DMEM). MG63 cell line selection is based on potential further applications of the AgNPs in bone tumor treatment. The culture plates populated with cells were incubated at 37 °C. The cell lines were treated with the following concentrations: 0.5 mg/mL, 1 mg/mL and 2 mg/mL of Ep–AgNPs, Es–AgNPs and Et–AgNPs for 24, 48 and 72 h. Each fraction of the studied samples was dissolved directly in the cell culture medium with a content of 2% dimethyl sulfoxide (DMSO) [34]. The MTT test was performed according to protocols described in literature and to ISO 10993-5 recommendations [35]. The absorbance of the formazan solution was measured at 570 nm, using a Tecan Sunrise plate reader spectrophotometer. An equivalent volume of isopropanol was used as reference. Cell viability was calculated using the following formula:

(1) $$\mathrm{Cell}\;\mathrm{viability}\;\%=\frac{\mathrm{Treated}\;\mathrm{absorbance}\;}{\mathrm{Control}\;\mathrm{absorbance}}\times 100$$

The absorbance determined in the experimental cultures was compared with that of the control cultures, in which the extract was not present (assays with vehicle only, 2% DMSO). All experiments were performed in triplicate [36,37].

Cell viability studies were carried out in triplicate (n = 3) for each experiment and analyzed by means of two-way ANOVA. A *p*-value of less than 0.01 was accepted as significant.

## 3. Results and Discussion

### 3.1. AgNPs Synthesis

In the synthesis of AgNPs using plant extracts, these general steps were followed: preparation of the extract and silver salt solutions, obtaining of the AgNPs by mixing the two solutions in different proportions, under certain pH conditions, and for different periods of stirring time. The results are available in the Supplementary Materials. The last steps were represented by the separation and purification of AgNPs, followed by confirmation of AgNPs formation through different methods [24,38].

First of all, the obtaining of AgNPs was monitored via color change. Therefore, after completion of the reaction, a change in color from yellow to brown was observed, which was attributed to the reduction of Ag^+^ to metallic nano-silver, Ag^0^. The modification in color can be observed in the images found in the Supplementary Materials. The successful synthesis of AgNPs was afterwards confirmed by SPR detection via UV-Vis spectrophotometry in the 400 to 500 nm range. The recorded peaks appeared at 435 nm for Ep-AgNPs, 442 nm for Es-AgNPs and 427 nm for Et-AgNPs.

Following all determinations, the conditions for the AgNPs synthesis were established as: AgNO_3_ concentration of 1 mM; extract:AgNO_3_ ratio 1:9 (*v*:*v*); pH 6; stirring time of 300 min.

To the best of our knowledge, the synthesis of AgNPs obtained using *E. pratense*, *E. sylvaticum* and *E. telmateia* has not been previously described in literature. However, different published studies reported that the SPR value of other biosynthesized AgNPs was located between 400 and 500 nm and any shift of this value may be attributed to the metabolites involved, which serve as reducing and stabilizing agents [39,40,41,42].

### 3.2. Physico-Chemical Characterization of the Obtained AgNPs

#### 3.2.1. TEM Analysis, DLS Characterization and Zeta Potential Determination

**Figure 1:**
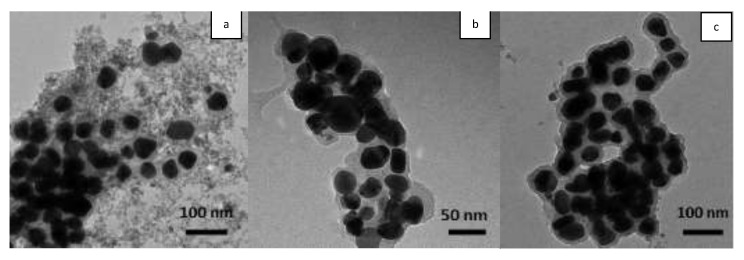
TEM images of Ep–AgNPs (**a**); Es–AgNPs (**b**); Et–AgNPs (**c**).

TEM analysis provides data on the shape and size of AgNPs, while the dimensional distribution (polydispersity index) and the hydrodynamic diameter of AgNPs diameter were determined by DLS. For all three samples, TEM images (Figure 1) showed that most AgNPs were well dispersed and with a spherical uniform morphology. The diameter of the silver core measured using TEM is approximately the same, with an average value of 50 nm. Moreover, the AgNPs are surrounded by a thin layer of material, suggesting that the nanoparticles are not in direct contact with each other, being encapsulated by biomolecules from extracts, which act as reducing and stabilizing agents. The hydrodynamic diameter measured by DLS depends on the nature of the capping agent and the obtained values are: 124.9 nm with a polydispersity index of 0.587 for Ep–AgNPs, 74.4 nm and 0.659 for Es–AgNPs and 314 nm with a polydispersity index of 0.623 for Et–AgNPs.

The Zeta potential values were −23.76 for Ep–AgNPs, −29.54 for Es–AgNPs and −20.72 for Et–AgNPs. These negative values show that the biomolecules found on the surface of AgNPs are negatively charged, causing rejection between nanoparticles, which means there is no aggregation and the obtained colloidal solution is stable [43,44]. However, long-term stability testing did not represent the focus of the current research.

#### 3.2.2. Fourier Transform Infrared Spectroscopy (FTIR)

**Figure 2:**
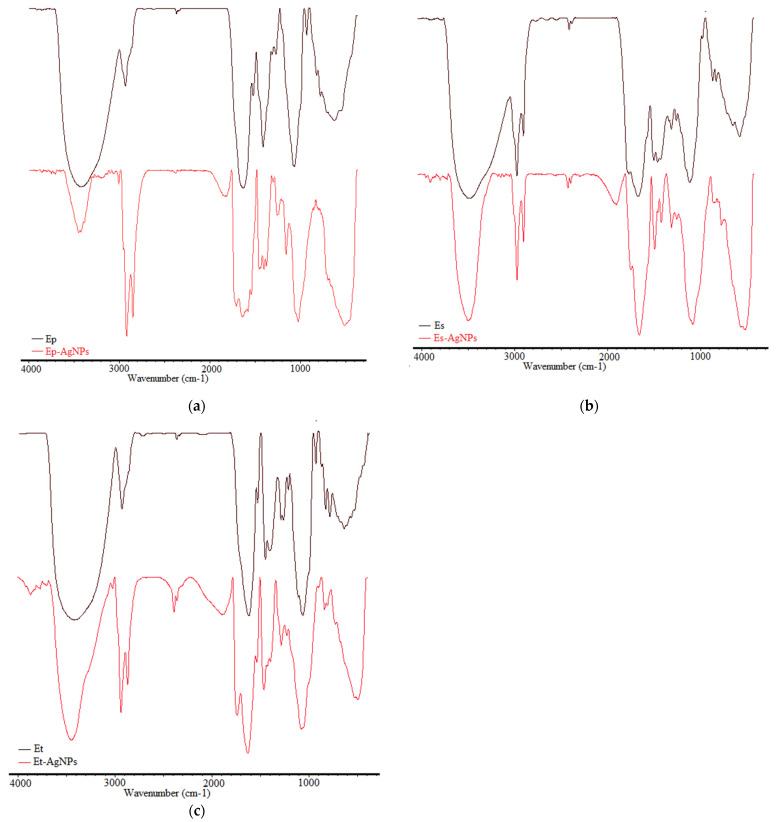
Comparative FTIR spectra of extracts and of corresponding AgNPs: Ep/Ep–AgNPs (**a**); Es/Es–AgNPs (**b**); Et/Et–AgNPs (**c**).

The functional groups of different biomolecules responsible for silver (Ag^+^) ions reduction and for capping and stabilizing of AgNPs were highlighted using FTIR analysis. The FTIR spectra of the extract and of the corresponding AgNPs are shown in a comparative manner in Figure 2.

To demonstrate the formation of AgNPs and to establish the chemical composition of the AgNPs surface, FTIR spectra of the extract and of the corresponding AgNPs were recorded. Therefore, the changes that took place in the absorption bands of the extract were compared to the absorption bands of the corresponding AgNPs.

For both extracts and AgNPs, the FTIR spectra showed clear peaks throughout the whole range of observation. The vibrations were assigned to the following absorption bands in the FTIR spectra of extracts, as seen in Table 1.

**Figure 3:**
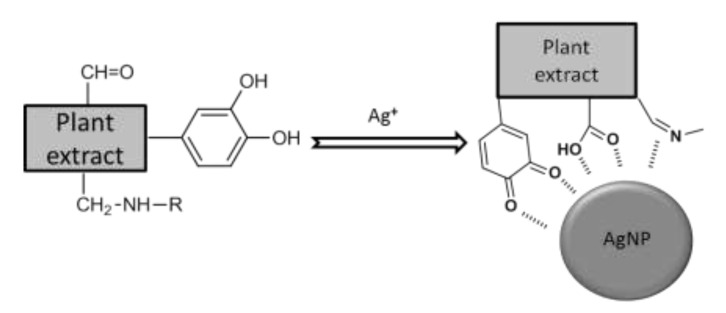
Schematic representation of the partial oxidation of the plant extract during AgNPs formation and interactions between functional groups and the silver surface.

Band shifts, splitting of absorption bands into smaller ones or appearance of new bands were observed for AgNPs. The emergence of bands in the FTIR spectrum of AgNPs can be explained by the attachment to the AgNPs surface of some groups of the extracts’ compounds. In all spectra of the samples containing AgNPs, a distinct appearance of vibrational bands is observed in the 1840–1870 cm^−1^ range, which are not found in the original extracts. These bands could be explained as a consequence of the interaction of molecules found in the extract with silver and could be attributed to carbonyl and imine double bonds resulting from the oxidation of organic compounds, especially those containing phenolic groups, followed by their interaction with the surface of silver. A possible representation of this process is shown in Figure 3.

Functional groups belonging to flavonoids, proteins, amino acids, sterols, carbohydrates and phenols found in the extracts contribute to the obtaining of AgNPs [19,47,48,49].

#### 3.2.3. Energy Dispersive Spectroscopic Analysis of AgNPs

**Figure 4:**
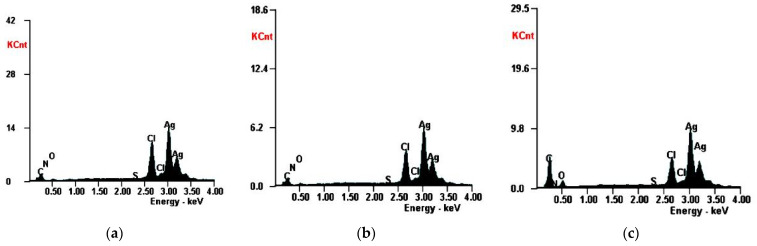
EDX spectra of Ep–AgNPs (**a**); Es–AgNPs (**b**) and Et–AgNPs (**c**).

In order to highlight the presence of silver, the EDX spectra of the synthesized nanoparticles were obtained and are presented in Figure 4.

The analysis of the recorded spectra revealed the presence of a peak at 3 keV, which is characteristic to metallic silver and is related to SPR [27,50]. Quantitatively, silver was found in a proportion of 60.68 (m %) for Ep–AgNPs, 63.08 (m %) for Es–AgNPs and 42.40 (m %) for Et–AgNPs. In addition to silver, characteristic peaks of carbon, oxygen and nitrogen could also be observed: 20.97% carbon, 1% nitrogen and 3.04% oxygen for Ep–AgNPs; 18.93% carbon, 1.52% nitrogen and 4.25% oxygen for Es–AgNPs and 39.47% carbon, 1.91% nitrogen and 9.42% oxygen for Et–AgNPs. The presence of elements other than silver proves that biomolecules from the extracts adhered to the surface of AgNPs [49].

#### 3.2.4. Phytochemical Evaluation of Phenolic Compounds from *Equisetum* Extracts Used for AgNPs Synthesis

Silver ions undergo a bioreduction process due to biomolecules present in the extract, being converted into colloidal silver that forms aggregates that will stabilize through the attachment to the surface of some biomolecules found in the environment. Therefore, given the fact that biomolecules present in the extract participate in the synthesis of AgNPs, the content of phenolic compounds in the initial extracts and in the supernatant obtained after separation of nanoparticles was determined. The results are shown in Table 2.

Generally, only around half of the initial content of phenolic compounds can be found in the supernatant obtained after separation of AgNPs, which clearly indicates the importance polyphenols play in the synthesis of such nanoparticles. The few studies previously done on other *Equisetum* species also point out that mostly hydroxyl groups found in polyphenols and flavonoids present in the plant are responsible for the reduction of the silver ion, followed by their oxidation to carbonyl groups [18,20,51].

### 3.3. Photocatalytic Activity of the Obtained AgNPs

In order to test the photocatalytic action of the obtained AgNPs, the degradation of a dye (*n*-methylated diaminotrifenylmethane or malachite green), which is used in various industries and can be found in aquatic and terrestrial ecosystems, therefore posing a potential danger to human health, was analyzed [29]. For this determination, the color change of a malachite green solution following treatment with the obtained AgNPs by magnetic stirring in sunlight was visually and spectrophotometrically observed. Visually, a clear decrease in color intensity from dark blue to light blue was noticed. The modification in color can be observed in the images found in Supplementary Materials. For the control, no color modification was observed.

**Figure 5:**
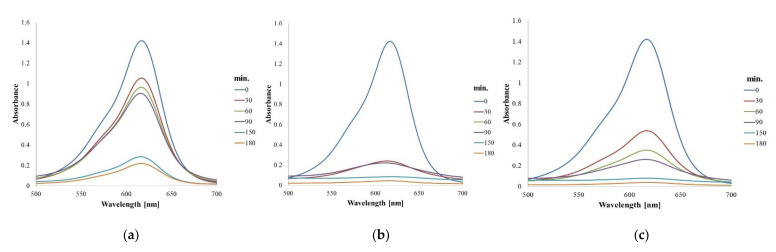
Photocatalytic degradation over time (UV-Vis spectra) observed for Ep–AgNPs (**a**); Es–AgNPs (**b**); Et–AgNPs (**c**).

Figure 5 shows the UV-Vis spectra proving the photocatalytic degradation that occurs after treatment with AgNPs by magnetic stirring under the influence of sunlight.

By spectrophotometric monitoring of the dye peak at 617 nm, a significant decrease in absorbance over time is observed in all cases, which demonstrates that there is a decrease in dye concentration due to degradation in the presence of AgNPs. The dye degradation percentage was calculated using the formula below.

(2) $$\mathrm{Dye}\;\mathrm{degradation}\;\%=\frac{C0-\mathrm{Ct}}{C0}\times 100$$

where: C0 represents the initial concentration of the dye and Ct the dye concentration after each period of light exposure [52].

For these samples, the percentage of dye degradation reached up to 84.54% for Ep–AgNPs, 96.89% for Es–AgNPs and 97.19% for Et–AgNPs.

The photocatalytic activity of AgNPs can be explained either through the excitation of electrons and their transition to a higher energy state due to the surface plasmon resonance effect, or through the absorption of UV radiation by AgNPs due to transitions between 4d and 5s orbitals with high energy electron generation. Furthermore, the excited electrons are accepted by oxygen molecules dissolved in the reaction medium which are transformed into superoxide anions (•O^2−^) and hydroxyl radicals (HO•), which destroy the structure of the dye, transforming it into smaller organic molecules, thus degrading it [38].

### 3.4. In Vitro Evaluation of the Antioxidant Activity of the Obtained AgNPs

The evaluation of the antioxidant action was done for the extracts obtained from the three species, as well as for the AgNPs synthesized using the previously mentioned plant extracts.

#### 3.4.1. Determination of the Ferrous Ion Chelating Capacity

**Figure 6:**
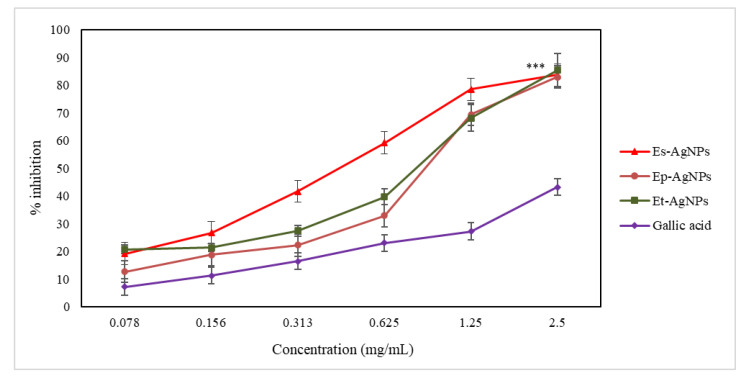
Graphical representation of the iron-chelating capacity (%) of AgNPs solutions compared to gallic acid. Values are expressed as the mean ± standard error of the mean from three independent experiments (n = 3). *** *p* < 0.0001 versus gallic acid (two-way ANOVA).

The ferrous ion chelating capacity is an important indicator for evaluating antioxidant activity. For silver nanoparticles, the capacity to block iron finds applicability in local anti-infective therapies, because AgNPs will block the access of microorganisms to iron that use it to defend against leukocytes [1,10]. Conjugation of iron inherently leads to a decrease in its ability to produce free radicals, and, consequently, iron chelation is also a measure of antioxidant capacity. The results obtained for this assay are presented in Figure 6.

For the determination of the ferrous ion chelating capacity, when comparing the EC_50_ values, it can be noticed that Es-AgNPs (86.86 ± 0.39 µg/mL) present an activity twice as intense compared to Ep-AgNPs (172.83 ± 0.54 µg/mL) and 1.8 times higher than that of Et-AgNPs (160.56 ± 1.72 µg/mL). EC_50_ represents the concentration at which nanoparticles show an inhibition/scavenger activity of 50%. The EC_50_ values are inversely proportional with the efficiency of the antioxidant activity.

Therefore, it can be stated that the ethanolic extracts that were used to obtain AgNPs have the ability to chelate ferrous ions, and the intensity of the action is much higher for the obtained nanoparticles. As only some of the active constituents present in the extracts participate in the formation of AgNPs, a clear ratio comparing the efficiency of the extracts with that of the nanoparticles cannot be calculated [2,10].

It has been shown that nanoparticles obtained using *E. sylvaticum* extract are almost twice as active compared to those obtained using *E. pratense* and *E. telmateia* extracts. This could be explained through the involvement of only some of the polyphenols found in the extract in the formation of AgNPs. Correspondingly, in the case of extracts, the best antioxidant activity was also observed for *E. sylvaticum* [53]. As shown in Figure 6, all AgNPs obtained from the *Equisetum* species showed a much more intense chelating capacity of the ferrous ion than gallic acid, which was used as standard.

#### 3.4.2. Determination of LOX Inhibition

**Figure 7:**
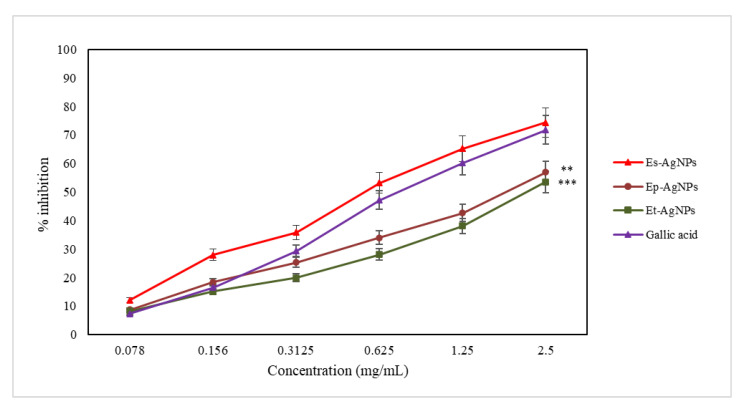
Graphical representation of the LOX inhibition capacity (%) of AgNPs solutions compared to gallic acid. Values are expressed as the mean ± standard error of the mean from three independent experiments (n = 3). ** *p* < 0.001, *** *p* < 0.0001 versus gallic acid (two-way ANOVA).

The active compounds present in the extracts inhibit 15-LOX (an enzyme of the oxidoreductase family) by blocking the oxidation of linoleic acid and reduce the absorbance measured at 234 nm [31]. The ability to inhibit LOX is another important indicator of the antioxidant action. The results obtained during this test are presented in Figure 7.

For the determination of LOX inhibition, Es-AgNPs (with an EC_50_ value of 9.14 ± 0.53 µg/mL) present a three times more intense activity compared to that of Ep-AgNPs (EC_50_ value of 29.88 ± 2.77 µg/mL) and four times higher than that of Et-AgNPs (EC_50_ value of 35.56 ± 2.68 µg/mL).

The difference in efficiency between AgNPs and the corresponding extracts is smaller compared to that obtained in the ferrous ion chelating capacity test, which implies the existence of much more complex mechanisms of inhibition compared to that of chelation. Among the obtained nanoparticles, the lowest efficiency regarding LOX inhibition can be seen for Et–AgNPs. The constituents of *E. sylvaticum* seem to possess the most important capacity of inhibiting LOX, taking into account results from previous studies [53] and the fact that only Es–AgNPs showed a higher efficiency compared to gallic acid.

#### 3.4.3. Determination of the Hydroxyl Radical Scavenging Capacity

The hydroxyl radical is synthesized in vivo or in vitro through Fenton and Haber-Weiss reactions, its synthesis being more intense under hypoxic conditions. It has a very high oxidizing capacity and can cause uncontrolled oxidation of DNA leading to genetic lesions, with the impairment of the ability to transmit accurate genetic information and of protein synthesis. Finally, the hydroxyl radical induces oxidation of proteins by modifying their spatial structure, thus affecting their biological functions [54]. The scavenging capacity of hydroxyl radicals is still an important indicator in the evaluation of antioxidant activity.

**Figure 8:**
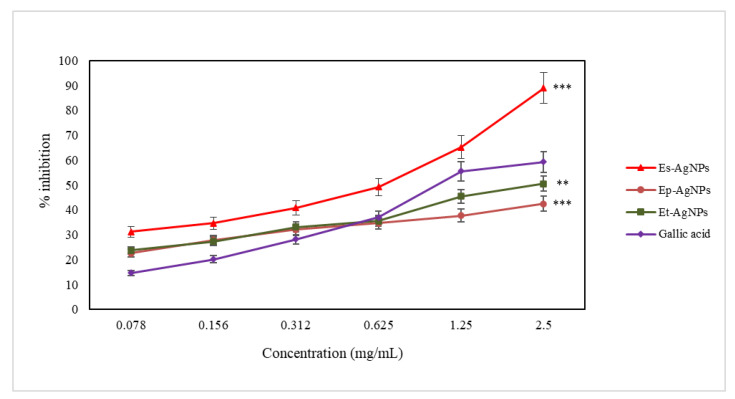
Graphical representation of the hydroxyl radical scavenging capacity (%) of AgNPs solutions compared to gallic acid. Values are expressed as the mean ± standard error of the mean from three independent experiments (n = 3). ** *p* < 0.001, *** *p* < 0.0001 versus gallic acid (two-way ANOVA).

The synthesized nanoparticles capture hydroxyl radicals and block the hydroxylation of salicylic acid, therefore reducing the intensity of the pink-purple coloration and of the absorbance measured at 562 nm. The obtained results are presented in Figure 8.

Regarding the determination of the hydroxyl radical scavenging capacity, the obtained results show that Es-AgNPs (60.79 ± 3.85 µg/mL) are 6 times more active compared to Ep-AgNPs (362.15 ± 4.29 µg/mL) and 3.3 times more active compared to Et-AgNPs (204.04 ± 24.21 µg/mL).

Hydrogen donor groups are required to neutralize the hydroxyl radical. It can be seen that Es–AgNPs showed the most significant hydroxyl radical scavenging capacity, comparable to that of the chosen standard. The lowest scavenging activity of the hydroxyl radical was recorded for Ep–AgNPs. The same order was observed in the case of extracts, as well [53]. In Figure 8, it can be seen that at a concentration of 0.625 mg/mL, the used standard (gallic acid), Ep–AgNPs and Et–AgNPs show approximately the same scavenging capacity. Although at low concentrations (0.078 mg/mL), the hydroxyl radical scavenging capacity (%) is the lowest for gallic acid, at higher concentrations (2.5 mg/mL) it increases, but it is still lower than that of Es–AgNPs. Neutralization of hydroxyl radicals depends mostly on the presence in the structure of scavanger compound(s) of groups capable of yielding hydrogen atoms, such as OH groups found in the structure of polyphenols.

#### 3.4.4. Determination of the Superoxide Anion Radical Scavenging Capacity

The superoxide anion radical is formed by reduction of molecular oxygen to accept only one electron, most often in the side reactions of the mitochondrial respiratory chain. Even if, from a chemical point of view, it is a rather weak oxidizing agent, it can generate hydroxyl radicals and singlet oxygen, which are very strong oxidants [55,56].

**Figure 9:**
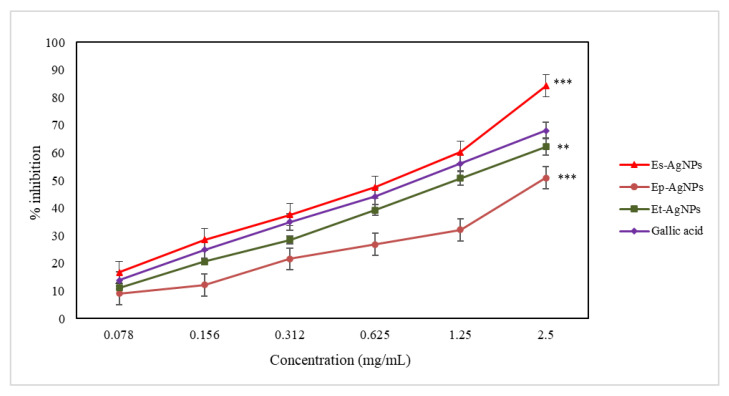
Graphical representation of the superoxide anion radical scavenging capacity (%) of AgNPs solutions compared to gallic acid. Values are expressed as the mean ± standard error of the mean from three independent experiments (n = 3). ** *p* < 0.001, *** *p* < 0.0001 versus gallic acid (two-way ANOVA).

The synthesized nanoparticles capture superoxide anions and reduce the synthesis of formazan with the reduction of the absorbance measured at 560 nm. The results obtained for this determination are presented in Figure 9.

Regarding the determination of the superoxide anion radical scavenging capacity, Es-AgNPs (178.80 ± 11.07 µg/mL) are at least 3 times more active compared to Ep-AgNPs (604.15 ± 28.00 µg/mL) and 1.6 more active compared to Et-AgNPs (298.36 ± 18.23 µg/mL).

Nanoparticles obtained from ethanolic extracts proved to be less effective in this test compared to the first three assays. However, it can be seen that in this case, as well as in the other performed tests, Es–AgNPs showed the highest scavenging capacity, higher than that of the used standard (gallic acid), while the weakest results were obtained for Ep–AgNPs. It can also be noted that the antioxidant efficiency of the standard is similar as value to that of Es–AgNPs and Et–AgNPs. Correspondingly, in the case of extracts, the highest antioxidant activity was also observed for *E. sylvaticum* and the lowest for *E. pratense*. If for the neutralization of hydroxyl radicals, only the presence of hydrogen donor groups in the scavenger molecule is required, for the neutralization of the superoxide anion radical, both hydrogen donor groups and functional groups capable of neutralizing the anion charge are required instead.

The antioxidant properties of AgNPs underline their possible use in reduction of local oxidative phenomena that occur in infectious processes.

### 3.5. In Vitro Evaluation of the Antitumor Action of the Obtained AgNPs

The present study aimed to evaluate the in vitro effects of AgNPs synthesized from ethanolic extracts of the three *Equisetum* species in terms of antitumor activity on cell lines showing osteosarcoma (MG63) [34,36].

**Figure 10:**
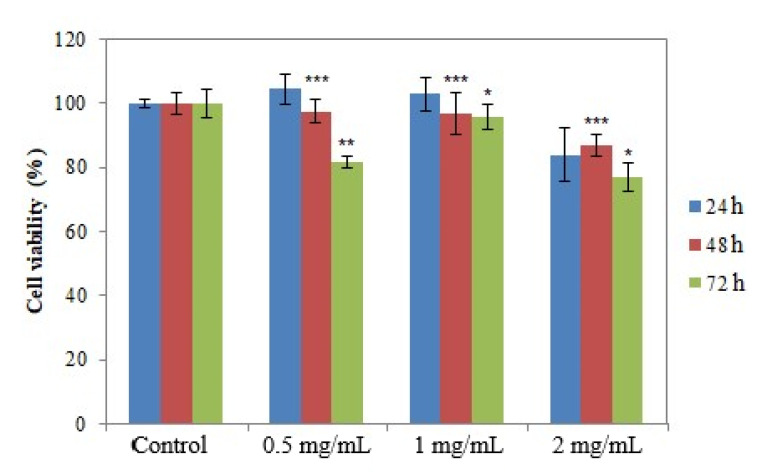
Cell viability determined using the MTT test for Ep–AgNPs. Values are expressed as the mean ± standard error of the mean from three independent experiments (n = 3). * *p* < 0.01, ** *p* < 0.001, *** *p* < 0.0001 versus control (two-way ANOVA).

**Figure 11:**
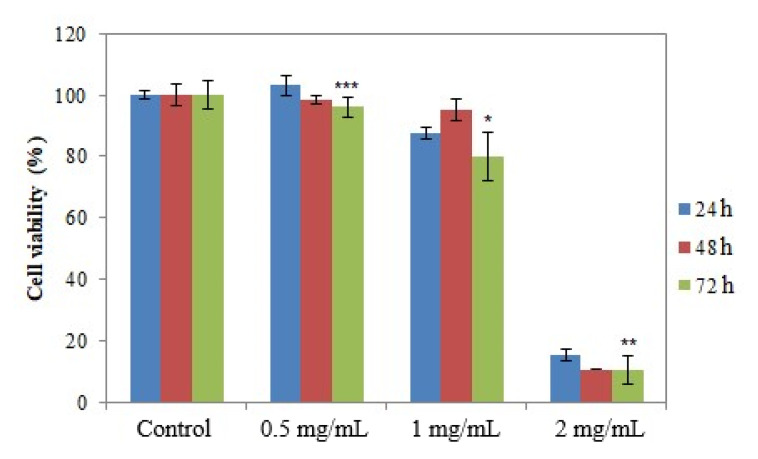
Cell viability determined using the MTT test for Es–AgNPs. Values are expressed as the mean ± standard error of the mean from three independent experiments (n = 3). * *p* < 0.01, ** *p* < 0.001, *** *p* < 0.0001 versus control (two-way ANOVA).

**Figure 12:**
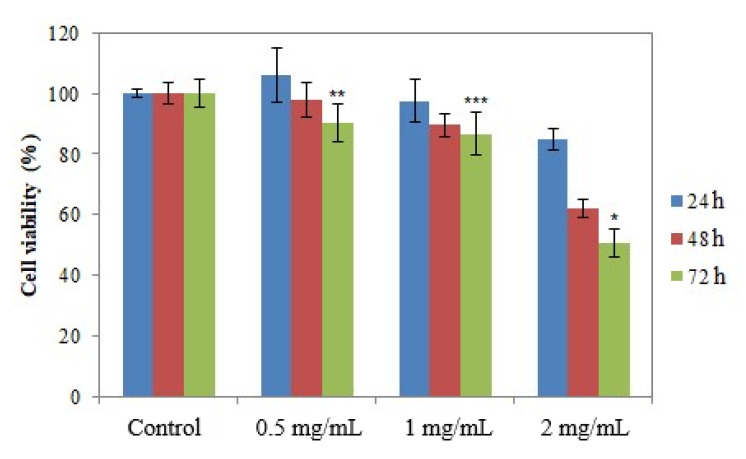
Cell viability determined using the MTT test for Et–AgNPs. Values are expressed as the mean ± standard error of the mean from three independent experiments (n = 3). * *p* < 0.01, ** *p* < 0.001, *** *p* < 0.0001 versus control (two-way ANOVA).

The presence of viable cells in the cultures incubated with the samples was assessed using the MTT method at 24, 48 and 72 h after incubation, respectively [35,37]. Figure 10, Figure 11 and Figure 12 show the results regarding cell viability reported for Ep–AgNPs, Es–AgNPs and Et–AgNPs, respectively.

As seen in Figure 10, Ep–AgNPs are not cytotoxic in the 0.5 mg/mL–1 mg/mL concentration range, with cell viability maintaining over 80% of the control value. For concentrations of 2 mg/mL, the cell viability decreases after a contact of 72 h to about 80%, which indicates that the LD_50_ cannot be calculated for this sample.

The cytocompatibility of Es–AgNPs at a concentration of up to 1 mg/mL can be observed in Figure 11. For a concentration of 2 mg/mL, cell viability drops dramatically below 15%, regardless of the contact time. It can be stated that the LD_50_ for this sample would be around 1.5 mg/mL. Consequently, the results obtained during this assay prove that silver nanoparticles obtained through green synthesis from *Equisetum* species could present applications in the treatment of such disorders. However, further studies focusing on the toxicity of the currently investigated samples on normal human cells must be performed in order to assess their selectivity.

Figure 12 shows that for Et–AgNPs no significant changes in cell viability for any of the studied concentrations appear. The increase in the contact time to 72 h and of the concentration to 2 mg/mL resulted in a decrease in cell viability of up to 52%. Under these conditions, it can be stated that LD_50_ for AgNPs synthesized using *E. telmateia* extract is slightly higher than 2 mg/mL.

After analyzing all figures, it can be easily seen that AgNPs synthesized using *E. sylvaticum* extract displayed the lowest cell viability, which corresponds to a high cytotoxicity. The determinations concluded that the lowest concentration that produced a change below LD_50_ for the analyzed cell lines is obtained for Es–AgNPs (2 mg/mL and 24 h). The highest cell viability was recorded for Ep–AgNPs, even at the highest studied concentration (2 mg/mL) and during the longest contact time (72 h), the result being slightly below 80%.

Similarly, in 2012, Costa-Rodrigues et al. studied the in vitro effect of a methanolic extract of *E. arvense* and concluded that it effectively reduced human osteoclast development and function, both in osteoclast precursor cell cultures and in osteoclastic and osteoblastic cell cultures [57].

One of the few cytotoxicity assays done on silver nanoparticles obtained from an *Equisetum* species, namely *E. arvense*, was performed on mammalian cells, MC3T3-E1 pre-osteoblast cells. Nanoparticles prepared at a low temperature exhibited non-toxic behavior. However, for the larger AgNPs, the non-toxic behavior of silver was extended to higher concentrations, proving that decreasing the size of AgNPs increases their cytotoxicity [18]. One other study carried out on AgNPs obtained using an aqueous extract of the same species proved their high cytotoxicity potential against HepG2 cells, that could be due to the induction of intracellular oxidative stress [58].

## 4. Conclusions

The spectrum of AgNPs obtained using the three *Equisetum* extracts showed the presence of the surface plasmonic band in the 400–500 nm range which proves the formation of nanoparticles. The DLS characterization and determination of Zeta potential showed that the biomolecules on the surface of the obtained AgNPs are negatively charged, causing a rejection between nanoparticles, which makes the obtained colloidal solution stable. The EDX characterization of AgNPs demonstrated that biomolecules found in the extracts adhered to the surface of AgNPs. Moreover, the FTIR characterization of AgNPs confirmed the attachment to their surface of functional groups such as H-O, C-H, C=O, C≡N, C≡C, COO^-^ and -CH=CH, mostly belonging to polyphenolic compounds.

The photocatalytic activity assay has shown that the synthesized AgNPs can be an ecological, fast and cost-effective choice for the degradation of organic dyes from the environment. Moreover, the obtained AgNPs also possess important antioxidant action, the best overall antioxidant capacity being observed for AgNPs obtained using *E. sylvaticum*. The in vitro antitumor assay led to a low cell viability value, corresponding to a high cytotoxicity for AgNPs obtained using *E. sylvaticum* ethanolic extract on the MG63 osteosarcoma cell line.

To the best of our knowledge, this is the first report on the synthesis of AgNPs obtained using either *E. pratense*, *E. sylvaticum* and *E. telmateia*. The used method is an eco-friendly alternative to classical synthesis methods and led to the obtaining of nanoparticles with important antioxidant, photocatalytic and cytotoxic activities.

## Figures and Tables

**Table 1:** Assignment of absorption bands in the FTIR spectra of extracts.

Ep	Es	Et	Vibration Type	Ref.
3433	3440	3427	H–O stretching intermolecular hydrogen bonding from alcohols or phenols	[45]
2929, 2364	2924, 2854	2927, 2362	C–H stretching vibrations of CH_3_ and CH_2_ (alkanes)	[45,46]
1629	1614	1616	Stretching vibrations C=O, C–N (amide I), asymmetrical stretching vibrations COO^-^	[46]
1516	1519	1523	Deformation vibrations *n*-H (amide II) and aromatic bonds	[46]
1408, 1307	1408, 1375	1444, 1402	C–O (amide) stretching vibrations and C–C stretching vibrations of phenyl groups, COO^-^ symmetric stretching vibrations and CH_2_ bond vibrations	[46]
1265	1259	1284, 1259	C–O stretching vibrations of alcohols, ethers, esters, carboxylic acids	[45]
1060	1060	1056	C–O and C–C stretching vibrations from carbohydrates	[46]
923–621	921–520	923–630	Bond vibrations C–H out of plane (alkenes)	[45,46]

**Table 2:** Content in phenolic compounds of the initial extract and of the supernatant obtained after separation of AgNPs.

Sample	Content in Phenolic Compounds (mg GAE/mL Sample) *	
	Initial Extract	Supernatant after Separation of AgNPs
Ep–AgNPs	1.1674 ± 0.001	0.5584 ± 0.003
Es–AgNPs	5.2478 ± 0.001	3.1163 ± 0.001
Et–AgNPs	9.2046 ± 0.002	4.6388 ± 0.002

* The results represent the mean value of triplicate quantification.

## Data Availability

Not available.

## Supplementary Materials

The supplementary materials are available online. Figure S1. Color monitoring of mixtures before and after reaction (I—Ep-AgNPs, II—Es-AgNPs, III—Et-AgNPs). Figure S2. The influence of different AgNO_3_ concentrations on the formation of AgNPs using *Equisetum* extracts: Ep(a), Es(b) and Et(c). Figure S3. The influence of the extract: AgNO_3_ molar ratio on the formation of AgNPs using *Equisetum* extracts: Ep (a), Es (b) and Et (c). Figure S4. The influence of the pH on the formation of AgNPs using *Equisetum* extracts: Ep (a), Es (b) and Et (c). Figure S5. The influence of the stirring time on the formation of AgNPs using *Equisetum* extracts: Ep (a), Es (b) and Et (c). Figure S6. Photocatalytic degradation of the dye solution in 180 minutes after treatment with AgNPs (I—Ep-AgNPs, II—Es-AgNPs, III—Et-AgNPs).

## Sample Availability

Not available.
