# Supplementary material for: In Vitro Antioxidant, Antitumor and Photocatalytic Activities of Silver Nanoparticles Synthesized Using Equisetum Species: A Green Approach
Source: Molecules. 2021 Dec 2;26(23):7325. doi: 10.3390/molecules26237325 (PMC8658848; doi:10.3390/molecules26237325)
Supplement: Supplementary file 1 [file molecules-26-07325-s001.zip › molecules-1484991-supplementary.pdf]

molecules

MDPI

Article

In Vitro Antioxidant, Antitumor and Photocatalytic Activities of Silver Nanoparticles Synthesized Using *Equisetum* Species: A Green Approach

Denisa Batir-Marin [1,†], Cornelia Mircea [2,†], Monica Boev [1], Ana Flavia Burlec [3,*], Andreia Corciova [3,*], Adrian Fifere [4], Alexandra Iacobescu [4], Oana Cioanca [5], Liliana Verestiuc [6] and Monica Hancianu [5]

[1] Department of Pharmaceutical Sciences, Faculty of Medicine and Pharmacy, Dunarea de Jos University, 800010 Galati, Romania; denisa.batir@ugal.ro (D.B.-M.); monica.boev@ugal.ro (M.B.)
[2] Department of Pharmaceutical Biochemistry and Clinical Laboratory, Faculty of Pharmacy, "Grigore T. Popa" University of Medicine and Pharmacy, 16 University Street, 700115 Iasi, Romania; cornelimircea@yahoo.com
[3] Department of Drug Analysis, Faculty of Pharmacy, "Grigore T. Popa" University of Medicine and Pharmacy, 16 University Street, 700115 Iasi, Romania
[4] Centre of Advanced Research in Bionanoconjugates and Biopolymers Department, "Petru Poni" Institute of Macromolecular Chemistry, 41A Grigore Ghica Voda Alley, 700487 Iasi, Romania; fifere@icmpp.ro (A.F.); iacobescu.alexandra@icmpp.ro (A.I.)
[5] Department of Pharmacognosy, Faculty of Pharmacy, "Grigore T. Popa" University of Medicine and Pharmacy, 16 University Street, 700115 Iasi, Romania; oana.cioanca@gmail.com (O.C.); mhancianu@yahoo.com (M.H.)
[6] Department of Biomedical Sciences, Faculty of Medical Bioengineering, "Grigore T. Popa" University of Medicine and Pharmacy, 16 University Street, 700115 Iasi, Romania; liliana.verestiuc@umfiasi.ro
* Correspondence: ana-flavia.l.burlec@umfiasi.ro (A.F.B.); acorciova@yahoo.com (A.C.)
† These authors contributed equally to this work.

Abstract: The ethanolic extracts of three *Equisetum* species (*E. pratense* Ehrh., *E. sylvaticum* L. and *E. telmateia* Ehrh.) were used to reduce silver ions to silver nanoparticles (AgNPs). The synthesized AgNPs were characterized using UV-Vis spectrophotometry, Fourier Transform Infrared Spectroscopy (FTIR), Energy Dispersive X-ray (EDX), Transmission Electron Microscopy (TEM) and Dynamic Light Scattering (DLS) measurements. FTIR data revealed the functional groups of biomolecules involved in AgNPs synthesis, such as O-H, C-H, C=O, C-O, and C-C. EDX spectroscopy was used to highlight the presence of silver, while DLS spectroscopy provided information on the mean diameter of AgNPs, that ranged from 74.4 to 314 nm. The negative Zeta potential values (−23.76 for Ep–AgNPs, −29.54 for Es–AgNPs and −20.72 for Et–AgNPs) indicate the stability of the obtained colloidal solution. The study also focused on establishing the photocatalytic activity of AgNPs, which is an important aspect in terms of removing organic dyes from the environment. The best photocatalytic activity was observed for AgNPs obtained from *E. telmateia*, which degraded malachite green in a proportion of 97.9%. The antioxidant action of the three AgNPs samples was highlighted comparatively through four tests, with the best overall antioxidant capacity being observed for AgNPs obtained using *E. sylvaticum*. Moreover, the biosynthesized AgNPs showed promising cytotoxic efficacy against cancerous cell line MG63, the AgNPs obtained from *E. sylvaticum* L. providing the best result, with a LD_{50} value around 1.5 mg/mL.

Keywords: *Equisetum pratense; Equisetum telmateia; Equisetum sylvaticum;* silver nanoparticles; green synthesis; photocatalytic activity; antioxidant capacity; cytotoxicity

Citation: Batir-Marin, D.; Mircea, C.; Boev, M.; Burlec, A.F.; Corciova, A.; Fifere, A.; Iacobescu, A.; Cioanca, O.; Verestiuc, L.; Hancianu, M. In vitro antioxidant, antitumor and photocatalytic activities of silver nanoparticles synthesized using *Equisetum* species: A green approach. *Molecules* **2021**, *26*, 7325. https://doi.org/10.3390/molecules26237325

Academic Editor(s): Davide Prosperi

Received: 13 November 2021
Accepted: 27 November 2021
Published: 2 December 2021

Publisher's Note: MDPI stays neutral with regard to jurisdictional claims in published maps and institutional affiliations.

Copyright: © 2021 by the authors. Submitted for possible open access publication under the terms and conditions of the Creative Commons Attribution (CC BY) license (http://creativecommons.org/licenses/by/4.0/).

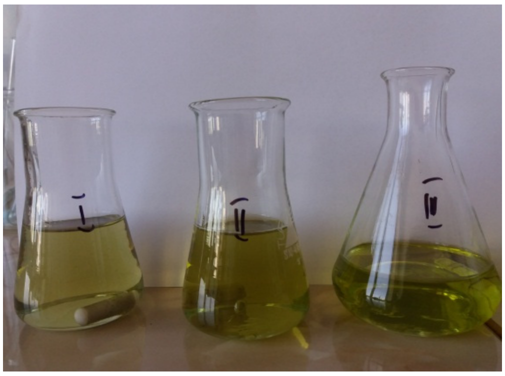

color before reaction

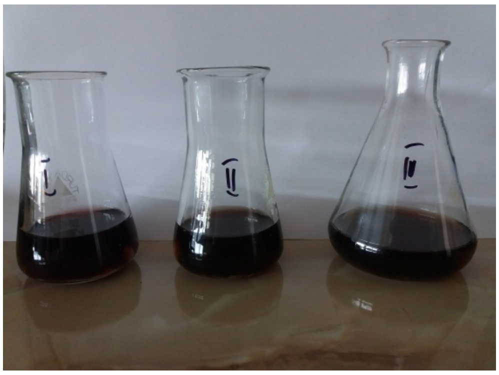

color after reaction

Figure S1. Color monitoring of mixtures before and after reaction (I – Ep-AgNPs, II – Es-AgNPs, III – Et-AgNPs).

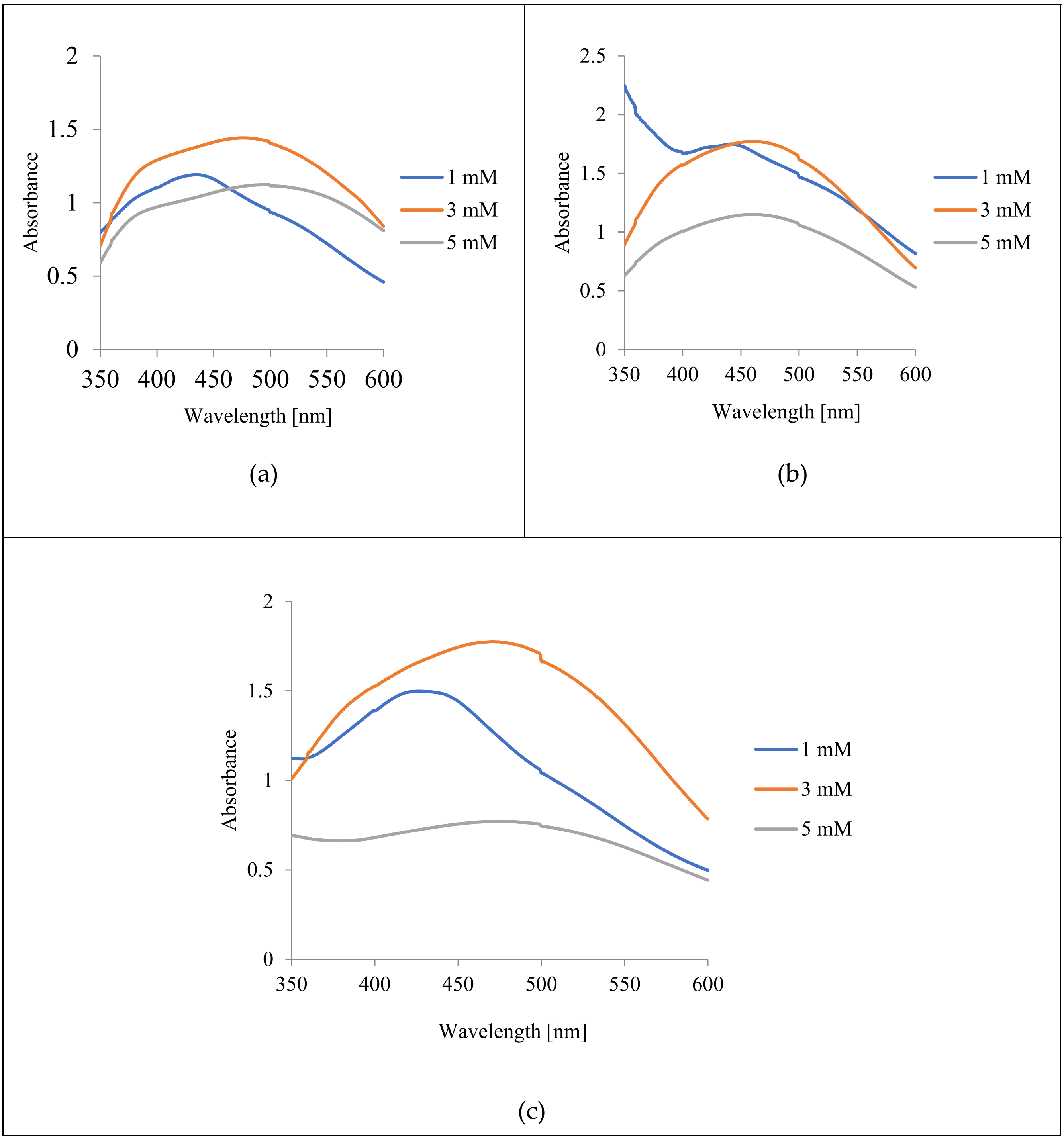

Figure S2. The influence of different $AgNO_3$ concentrations on the formation of AgNPs using *Equisetum* extracts: Ep (a), Es (b) and Et (c).

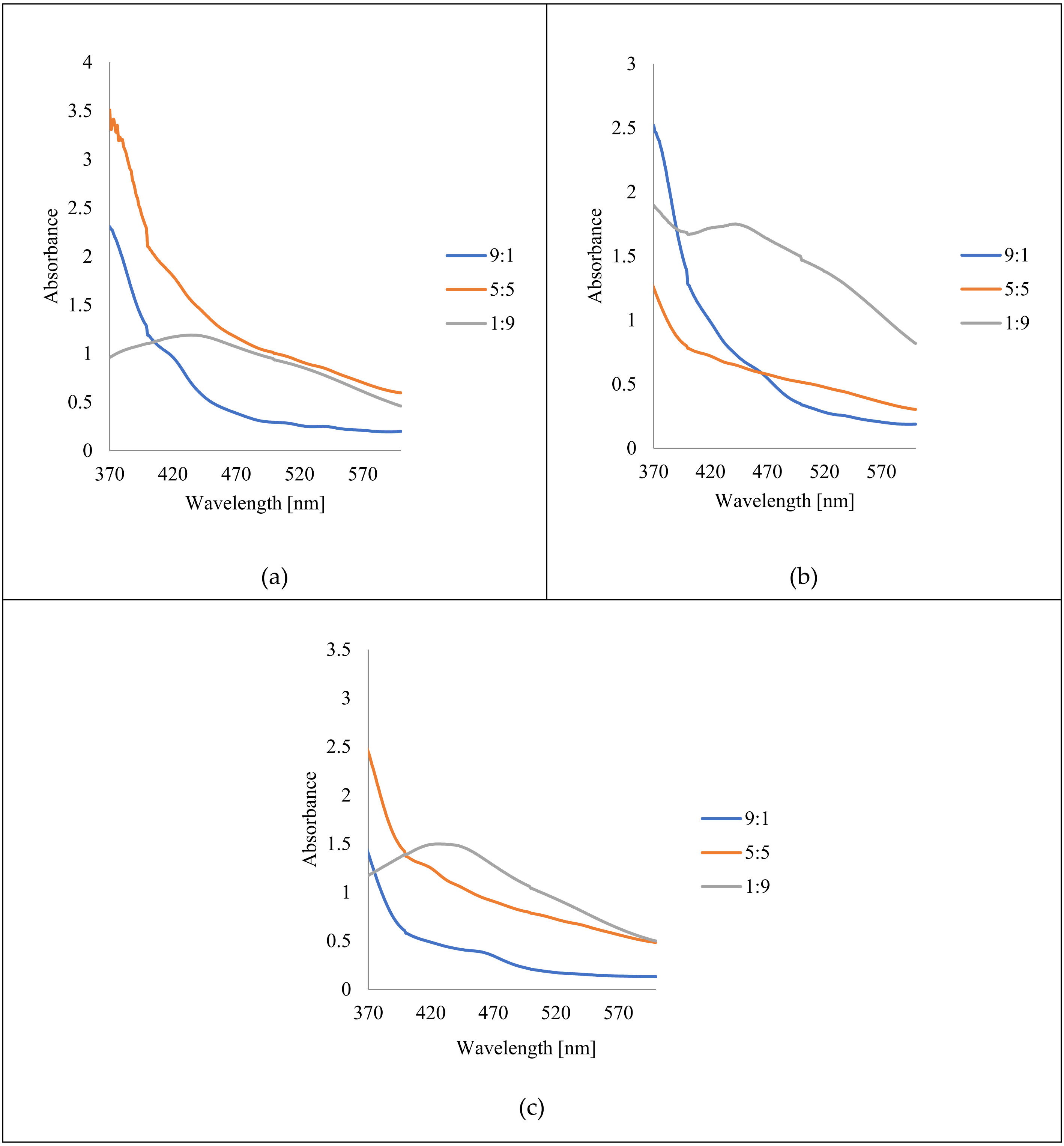

Figure S3. The influence of the extract:$AgNO_3$ molar ratio on the formation of AgNPs using *Equisetum* extracts: Ep (a), Es (b) and Et (c)

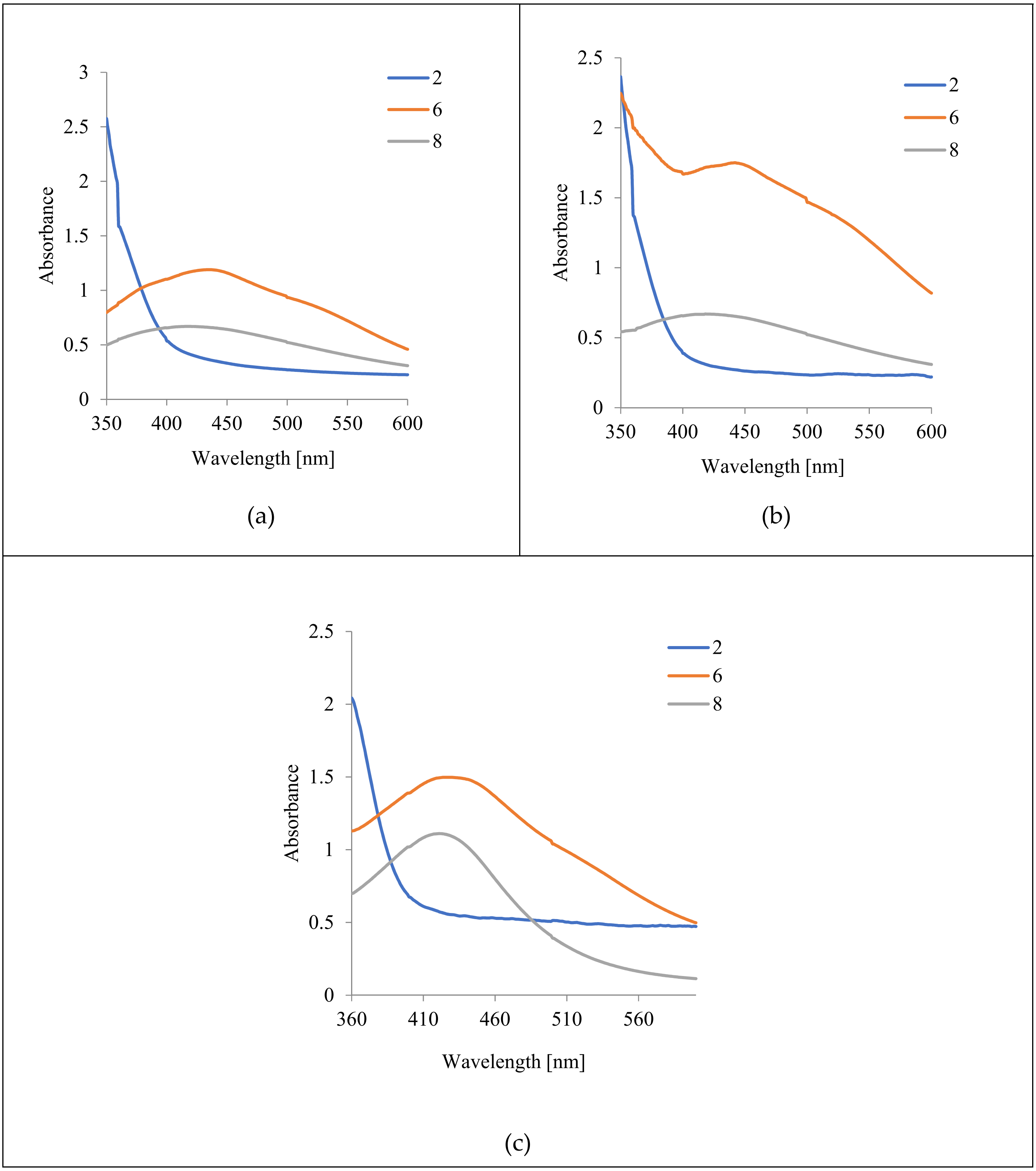

Figure S4. The influence of the pH on the formation of AgNPs using *Equisetum* extracts: Ep (a), Es (b) and Et (c).

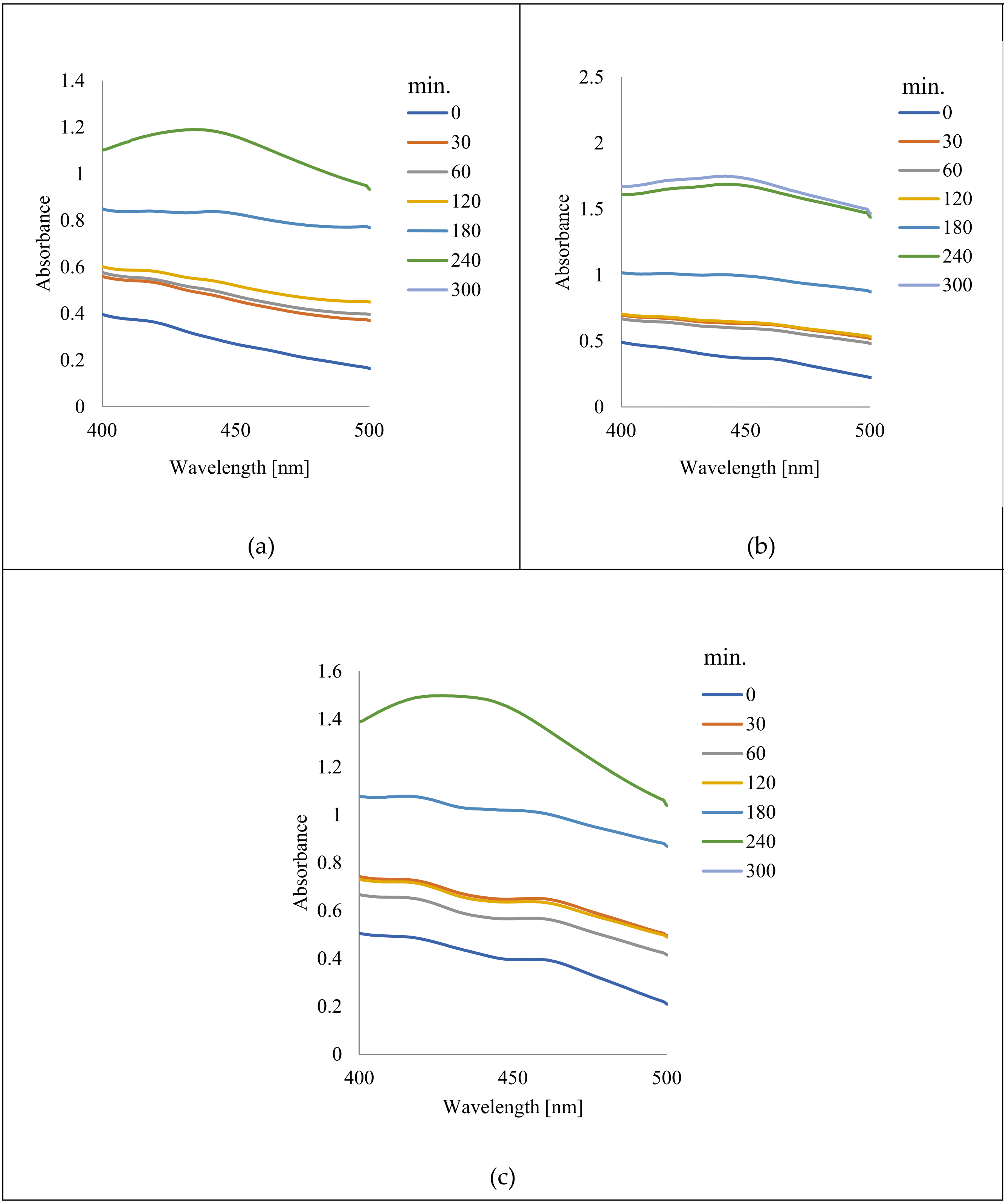

Figure S5. The influence of the stirring time on the formation of AgNPs using *Equisetum* extracts: Ep (a), Es (b) and Et (c).

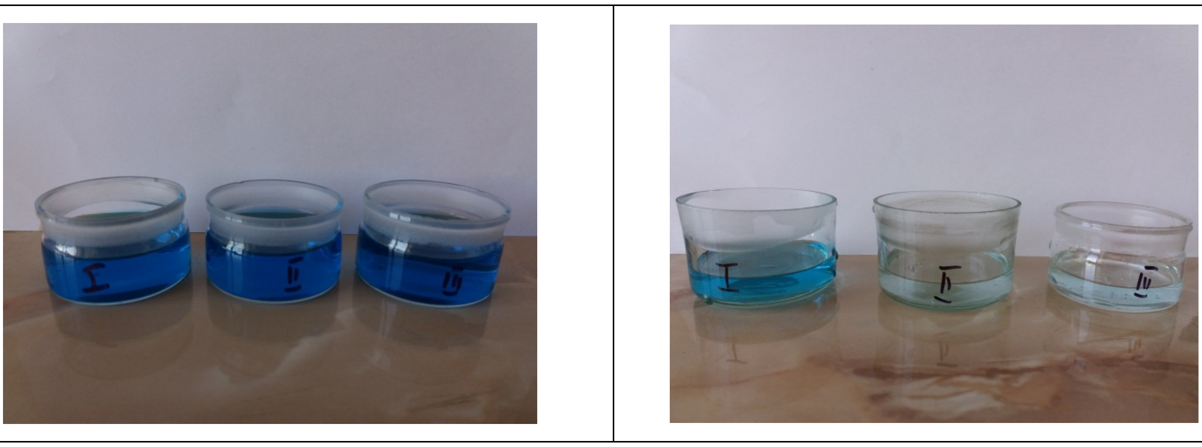

Figure S6. Photocatalytic degradation of the dye solution in 180 minutes after treatment with AgNPs (I – Ep-AgNPs, II – Es-AgNPs, III – Et-AgNPs).
